# Personality descriptions influence perceived cuteness of children and nurturing motivation toward them

**DOI:** 10.1371/journal.pone.0279985

**Published:** 2023-01-18

**Authors:** Reina Takamatsu, Takashi Kusumi, Hiroshi Nittono

**Affiliations:** 1 Graduate School of Education, Kyoto University, Kyoto, Japan; 2 Graduate School of Human Sciences, Osaka University, Osaka, Japan; Oslo New University College, NORWAY

## Abstract

The current empirical evidence regarding the effects of personality on physical attractiveness is limited to adult faces. In two preregistered studies, we demonstrated that personality descriptions influenced perceived cuteness, warmth, competence of young children, and female adults’ nurturing motivation toward them. Study 1 showed that participants rated children accompanied by positive personality descriptions as cuter, friendlier, and more intelligent than their initial ratings. Negative personality descriptions reduced perceived cuteness in children, which in turn reduced nurturing motivation. Study 2 showed that negative personality descriptions consistently reduced perceived cuteness and warmth ratings after manipulation, regardless of the initial level of perceived cuteness. After one week, cuteness and warmth ratings in the positive personality condition tended to return to their initial ratings. However, the effect of negative personality descriptions on cuteness ratings persisted for all children. Together, our findings suggest that female adults’ perception of cuteness and nurturing motivation are induced not only by children’s appearance but also their personality.

## Introduction

Visual cues provide powerful information for navigating social life. People frequently rely on appearance to form their first impressions of others [1,2], even outside their consciousness [3]. Both positive and negative physical attributes influence impression formation and future interactions. Attractive individuals are evaluated as friendly, kind, and capable [4]. They tend to lead to positive social interactions in multiple situations such as dating, job interviews, and negotiation [5]. In contrast, unattractiveness often leads to unfavorable social perceptions such as poor ratings of sociability, altruism, and intelligence [6]. Furthermore, negative perceptual information, including physical unattractiveness, has a more significant influence on evaluations than positive information [7–9], suggesting that those individuals expend more efforts to initiate a relationship than attractive others. Thus, whether people are aware or not, the physical (un)attractive stereotype (beauty is good; ugliness is bad) perseveres, and unattractiveness has more potent effects on social cognition than attractiveness does.

However, nonphysical traits may weigh more than appearances in long-term intimate bonds [10–14]. Both women and men, children, and adults consider personality traits more important than physical attractiveness in choosing a friend and partner [11,15]. As relationships grow, people pay more attention to and prioritize nonphysical characteristics such as respect, likability, and shared goals, rather than physical attractiveness [16,17]. An unattractive face becomes attractive after people learn about an individual’s favorable characteristics [18]. These findings suggest that non-physical traits play a pivotal role in nourishing intimate relationships, while physical attractiveness may be useful for initiating social exchanges.

Here, the following question arises: Do nonphysical traits influence adult ratings of children’s physical appearance and the quality of the parent-child relationship? A human child spends many hours in close physical proximity to their caretaker, and the parent-child relationship lasts for a lifetime [19]. Based on previous findings on personality and enduring partnerships [11], personality should also influence a parent-child relationship, especially parental attachment to the child. Personality emerges early in development, and parents can discern toddlers’ personality traits in detail [20]. Nonphysical traits, such as a child’s difficult temperament, developmental delays, and behavioral problems, elevate parental stress and undermine the motivation for caregiving [21–27]. In the next two sections, we review the effect of infantile characteristics of babies and young children on the perception of cuteness and nurturing motivation and discuss how personality can affect them.

### Baby schema effect, perceived cuteness, and nurturing motivation

For human infants to survive, parenting systems have evolved to elicit care from adults. Infantile facial features of babies and toddlers, such as big eyes, short nose, narrow chin, and high forehead, are perceived as cute, grab attention, induce pleasant feelings, and promote caregiving behaviors [28–33]. Infantile (Kindchenschema) characteristics related to the perception of vulnerability, innocence, and the need for care activate the parenting system, known as the baby schema effect [30,33–37]. Brain areas associated with social-emotional, reward, and motivational processing are activated when watching visual cues of baby cuteness [34,38,39]. To reiterate, infants’ and young children’s infantile physical characteristics are visually attractive and effective in inducing nurturing motivations and behaviors in adults.

Cuteness is a strong signal for care; however, this may imply that the absence of cuteness cues may negatively affect the parent-child relationship. Babies with facial malformations (e.g., cleft lip) are perceived as less cute than unaffected babies [40,41]. Mothers of infants with cleft lips are less responsive to their child in mother-child interactions, which leads to delayed social and cognitive development in the first two months of life [42]. Moreover, facial malformations disrupt the neural processing of the baby schema effect, as evidenced by reduced activation of the orbitofrontal cortex [43].

As reviewed above, the baby schema effect is robust. However, there are a few intriguing questions that the baby schema effect does not address. For example, why do they fail to operate under certain circumstances? Large-scale data indicate that in contrast to the innate parenting system (i.e., the baby schema effect), children under three are the most vulnerable to domestic violence [44]. The weak baby schema effect may be a disadvantage, but not detrimental, for a parent-child tie. Children with craniofacial anomalies are not particularly susceptible to punitive discipline at home [45]. Risk factors for child maltreatment include largely nonphysical factors rather than facial deformities [46]. Moreover, the human child requires many years of parental care and protection, but the baby schema effect lasts until age 3–4 [47,48]. Although the baby schema effect provides an evolutionary account of the perception of cuteness and caregiving, it may not be sufficient for creating a holistic picture of nurturing motivation in parenting.

### Effects of personality on perceived cuteness and nurturing motivation

One study [49] investigated whether experimentally assigned temperaments influence the perceived cuteness of babies and female adults’ motivation to view their faces. After learning about babies’ temperaments, female participants rated babies with easy temperaments as cuter than the initial ratings. Moreover, they expended more effort on viewing babies with easy temperaments than those with difficult temperaments [49]. These findings suggest that parents may feel more attached to a baby with happy dispositions than to a child with a negative temperament.

A child’s nonphysical traits may be important for the perception of cuteness and parental nurturing motivation, particularly in early childhood. Along with the research discussed above [49], available evidence supports this prediction. For instance, women and men rated 3-year-old and 9-year-old children who expressed immature thinking with a supernatural explanation (e.g., animism, finalism) as cuter, friendlier, and more endearing than children who expressed mature thinking [50]. The quality of the child-parent relationship is influenced by child nonphysical traits, such as temperament [22,24], behavioral problems [23], and developmental issues [25,26]. Moreover, in early childhood, personality traits become more visible than ever, and parents can report the profile of their children’s personality traits [20,51]. Given the ongoing nature of parent-child relationships, nonphysical traits, such as personality, should become significant over time for parents to perceive cuteness and nurture their preschool children.

#### Stereotype content of warmth and competence

According to the stereotype content model, warmth and competence are essential dimensions of social cognition as they can explain how people evaluate and treat others across contexts [52]. For example, people try to protect others who look warm, while they think it is morally acceptable to exclude others who look competent but not warm [53]. Perceived warmth, but not competence is related to the perception of vulnerability and a need for care [52]. We included warmth and competence to examine how personality information about young children influences the two dimensions of social cognition. For the warmth ratings, we included “being nice to others” as a central trait in the warmth dimension because, for young children, a personal attribute of a good friend is being nice to others [54,55].

### Current research

We are not aware of any studies that have investigated whether personality descriptions influence the perceived cuteness of young children. Do personality descriptions change an adult’s perception of cuteness in the child and their motivation for caretaking? Additionally, we were interested in the effects of personality descriptions on young children’s perceived friendliness and intelligence (i.e., warmth and competence of the stereotype content model) [52]. Furthermore, we also measured perceived infantile characteristics to test a model that predicts nurturing motivation. The baby schema effect has been based on the assumption that the perception of child’s infantile characteristics activate the human parenting system [28–33]. To directly test this model, we included the perceived infantility measure.

In two studies, we investigated the effects of personality descriptions on young children’s perceived cuteness, warmth, competence, and nurturing motivation. Study 1 examined the effects of personality description on young children’s perceived cuteness, warmth, competence, and nurturing motivation. Study 2 examined whether the initial level of physical cuteness in children modulates the above effects of personality descriptions, and whether the effects last after one week. One of the limitations of previous studies investigating the effect of personality information on physical attractiveness is that they have only considered its short-term effects. People may form an impression of others quickly, but in real-life settings, we may modify or forget the initial impression after interacting with the person. To investigate the long-term effects of personality, we included a follow-up survey in Study 2.

### Hypotheses

We pre-registered the following hypotheses: First, based on previous research on adults [11,17], we hypothesized that positive personality descriptions would increase young children’s perceived cuteness, warmth, and competence (H1a). In contrast, negative personality descriptions reduced young children’s perceived cuteness, warmth, and competence (H1b). As in a previous study [6], we also included the competence dimension because unlike warmth, intelligence is less influenced by physical attractiveness than sociability [4].

Second, we hypothesized that perceived cuteness would mediate the link between type of personality description and nurturing motivation toward young children (H2). Specifically, we expected higher perceived cuteness to mediate the association between positive personality descriptions and nurturing motivation. By contrast, lower perceived cuteness was expected to mediate the link between negative personality descriptions and reduced nurturing motivation. This hypothetical model was driven by the findings of previous research showing that infant faces with high baby schema increase the incentive for caregiving [30,34,38,56].

Thirdly, we hypothesized that the effect of personality information on perceived cuteness, warmth, and competence will have long-term effects (H3). To test H3, participants in Study 2 rated perceived cuteness, warmth, and competence of children again after one week.

## Study 1

### Materials and methods

Reports of all measures, experimental manipulations, visual stimuli, pre-calculated sample size, exclusion criteria, data files, and analysis codes are available for download at https://osf.io/h6rfv/?view_only=1e29b40ef8bb4301bd3fcaa44ee1d79c. The proposed studies are registered at https://archive.org/details/osf-registrations-ucmzy-v1. All studies were approved by the ethical review board (ERB) of the Kokoro Research Center, Kyoto University (3-P-6). Written consent was provided by all participants.

We conducted an online pilot study to select 18 facial images of young children with low, moderate, and high levels of physical cuteness. Participants (*N* = 54) rated the physical cuteness of 40 images of faces of female and male children with neutral expressions (1 = *not at all cute* to 10 = *very cute*). The age range of the children was 3–4 years. Based on the mean rating (5.95, *SD* = 1.85), 18 images (three boys and three girls) were selected for high cuteness (more than the mean +1*SD*; *M* = 7.25), low cuteness (less than the mean −1*SD*; *M* = 4.84), and moderate cuteness (in between them; *M* = 5.66).

Another online pilot study was conducted to select 12 personality descriptions that delineate the desirable or undesirable personality traits of young children. Given that child personality traits can be organized into the Big Five model across cultures [51], the personality descriptions were based on the five traits (openness to experience, conscientiousness, extraversion, agreeableness, neuroticism). We also included culture-specific personality traits of young children, such as *amae* [57]. These descriptions included the child’s profiles at home and at kindergarten because the Japanese may behave differently depending on the context [58]. Participants (*N* = 70) read 20 descriptions of young children’s personality traits and rated the extent to which the descriptions were desirable (1 = *very undesirable* to 6 = *very desirable*). Based on the average desirability rating (3.72, *SD* = 0.98), six desirable (more than the mean + 1*SD*; *M* = 4.82) and six undesirable personality descriptions (less than the mean − 1*SD*; *M* = 2.48) were identified. Desirable personality traits included: “helps his/her mother voluntarily,” “tries to cheer up his/her crying friend,” and “is good at drawing and shows her drawings to others proudly.” Undesirable personality traits included: “cuts in line because he/she wants to play in the swings,” “eats all the snacks he/she should share with his/her brother,” and “often fights and hits his/her friend.” The details of the pilot studies are provided in the S1 File.

#### Power analysis

G*Power (Version 3.1) [59] specified that a sample size of 72 would be needed to obtain 80% power to detect a small effect (*f* = .14) with an alpha of .05 in a repeated-measures analysis of variance (ANOVA).

#### Participants

We recruited 72 Japanese female adults between the ages of 21 and 48 years (*M*_*age*_ = 36.3, *SD* = 6.7) who had an active worker account on CrowdWorks. Of these, 23 participants were parents (31.9%). We advertised the recruitment of female adults over the age of 20 years because adolescents are less sensitive to cuteness cues than adults [60]. In addition, this is the first investigation to study the effect of personality traits on young children’s perceived cuteness. Our primary focus was not on gender differences in the perceptions of cuteness. Previous findings on gender differences in sensitivity to cuteness have been mixed [43]. For these reasons, we recruited only women aged 20 years or over. All participants had a visual acuity in the normal range.

#### Study design and procedure

This study employed a within-subjects design. The within-subject factors were time (pre-evaluation, post-evaluation) and personality descriptions (positive, negative, control [no description]). In the positive personality condition, participants saw two children’s faces with descriptions of two desirable personality traits each. In the negative personality condition, participants saw other two children’s faces with descriptions of two undesirable personality traits each. The personality descriptions were not presented in the control condition. As in previous studies [11], this condition was included as a control. We used six faces from children with moderate levels of physical cuteness (three girls and three boys). These faces were randomly assigned to three conditions in each participant. For exploratory purposes, we also included child gender as a within-factor (S2 File).

Before the main procedure, participants read 15 personality descriptions of young children and rated the extent to which they were desirable (1 = *very undesirable* to 6 = *very desirable*). The presentation order of descriptions was randomized across participants.

*Pre-evaluation*. Participants viewed six pictures of young children for five seconds in a counterbalanced order. After viewing each image, they evaluated a) perceived cuteness (cute, adorable, endearing), b) warmth (good-natured, friendly, compassionate), c) competence (intelligent, capable, bright), and d) perceived infantile characteristics (three items: vulnerable, naïve, caretaking) [35] in a randomized order. Responses were recorded on a 10-point scale (from 1 = *strongly disagree* to 10 = *strongly agree*).

*Distraction task (5 minutes)*. Immediately after the pre-evaluation, the participants were asked to complete simple math quizzes (completing an equation by filling an empty box) and a visual search task (counting hidden triangles in a picture).

**Fig 1:**
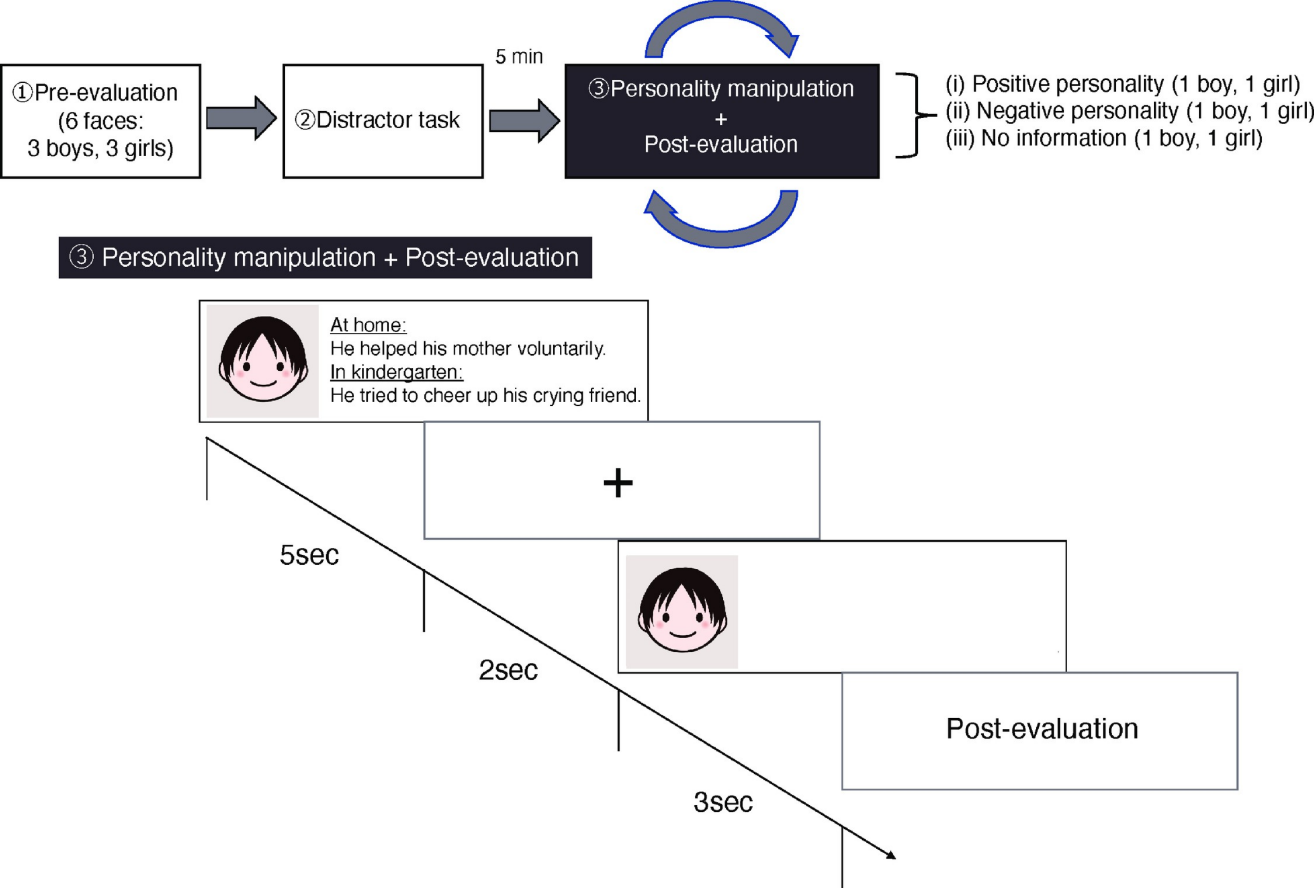
Experimental procedure. The personality description here is an example of positive personality traits.

*Personality manipulation and post-evaluation*. Fig 1 shows the procedure. Participants viewed the same picture of children with a personality description (no description in the control condition) in a counterbalanced order. In the positive personality condition, positive personality descriptions were presented with a child’s face for five seconds. In the negative personality condition, negative personality descriptions were presented with a child’s face for five seconds. In the control condition, no personality description was presented, and only the child face was presented for five seconds. After a fixation mark, the child’s face was presented again without personality descriptions for three seconds. This was to keep the appearance of a stimulus to be judged constant across the positive, negative, and control conditions. Then, the screen automatically turned to the next page for post-evaluation questions. Again, participants completed the same set of items measuring cuteness, warmth, competence, and perceived infantile characteristics in a randomized order.

In the post-evaluation phase, participants completed additional two items for measuring nurturing motivation toward children (“I want to protect the child,” “I feel tenderness toward the child”) [61]. Responses were recorded on a 10-point scale (from 1 = *strongly disagree* to 10 = *strongly agree*).

#### Statistical analysis

We computed Cronbach alphas for cuteness, warmth, competence, infantile characteristics ratings based on the ratings of all subjects on all images separately for pre- and post-evaluations. For the nurturance motivation, we computed Pearson correlation coefficients of the two items.

To test H1a and H1b, a series of 2 (time: pre-evaluation, post-evaluation) × 3 (personality descriptions: positive, negative, control) repeated-measures ANOVA were performed. The dependent variables were cuteness, warmth, competence, and infantile characteristics. The significance level was set at .05. For additional analyses, we tested H1a and H1b, using the generalized linear mixed-effect models (GLMM; S3 File). The random intercepts by participants, participants × time, participants × personality descriptions, and stimuli variability were included in the models. The GLMM results were comparable to those of ANOVAs.

To test H2, we ran multi-categorical mediation analyses using the PROCESS macro [62] with experimental conditions as a multi-categorical predictor, perceived cuteness as a mediator, and nurturing motivation as an outcome variable. We used an indicator coding scheme as recommended in [63]. We assigned 0 to the control condition to make it the reference category. In the first set of dummy coding (D_1_), the positive and negative personality conditions were coded as 1 and 0, respectively. In the second set of dummy coding (D_2_), the positive and negative personality conditions were coded as 0 and 1, respectively. We used 5,000 bootstrapped samples to estimate the bias-corrected 95% confidence intervals around the indirect effect. Given that parents are more sensitive to cuteness cues than are non-parents [64], for an exploratory purpose, parental status was entered as a covariate (S4 File). A mediation model is significant if the bias-corrected 95% confidence interval (CI) does not include zero [65]. After confirming an indirect effect, we examined the residual effect of positive/negative personality descriptions on nurturing motivation to see whether the mediation is partial or full.

### Results

#### Preliminary analyses

Manipulation of personality descriptions was successful. Consistent with the results of the pilot test, the main effect of personality descriptions on the desirability rating was significant, *F*(2, 142) = 669.09, *p* < .001, η_p_^2^ = .90. The post-hoc multiple comparisons (*p*-values corrected by the Bonferroni method) revealed that participants rated positive personality descriptions (*M* = 8.60) as more desirable than negative (*M* = 1.18), *t*(71) = 33.04, *SE* = .14, *p* < .001, *d* = 1.65; and neutral ones (*M* = 5.41), *t*(71) = 19.30, *SE* = .13, *p* < .001, *d* = 1.40.

Cronbach’s alphas were .94−.97 for cuteness, .83−.93 for warmth, .83−.94 for competence, and .53−.70 for perceived infantile characteristics. The Pearson correlation coefficients of the two items (“I want to protect the child,” “I feel tenderness toward the child”) were .72−.91.

#### Primary analyses

Changes in cuteness, warmth, and competence ratings from pre- to post-evaluations are shown in Table 1.

**Table 1:** Changes in cuteness, warmth, and competence ratings from pre-evaluation to post-evaluation (Study 1).

	Personality information					
	Positive		No information		Negative	
	Δ	*p*, 95% CI	Δ	*p*, 95% CI	Δ	*p*, 95% CI
Cuteness	1.13	< .001 [0.80, 1.47]	−0.05	.615 [−0.28, 0.18]	−2.20	< .001 [−2.69, −1.72]
Warmth	2.02	< .001 [1.69, 2.36]	−0.11	.261 [−0.34, 0.19]	−2.52	< .001 [−2.97, −2.06]
Competence	1.27	< .001 [0.94, 1.60]	−0.44	.017 [−0.69, −0.20]	−2.06	< .001 [−2.55, −1.56]

*N* = 72. Δ = changes in the ratings from pre-evaluation to post-evaluation.

#### Effects of personality descriptions

**Fig 2:**
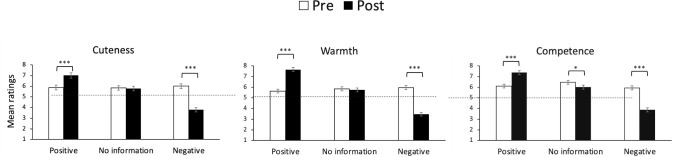
Cuteness, warmth, and competence ratings before/after personality manipulation (Study 1). Error bars indicate standard error. The dotted lines indicate the midpoints. *p < .05, ***p < .001.

Fig 2 presents cuteness, warmth, and competence ratings before and after personality manipulation. The results supported H1a, which is that positive personality descriptions increase perceptions of cuteness, warmth, and competence. The results also supported H1b, which is that negative personality descriptions reduce young children’s perceived cuteness, warmth, and competence.

*Cuteness*. There was a significant main effect of time on cuteness of child faces, *F*(1, 71) = 11.69, *p* = .001, η_p_^2^ = 0.14. We found a significant interaction effect between time and personality description, *F*(2, 142) = 89.26, *p* < .001, η_p_^2^ = .56. Post-hoc comparisons (Bonferroni) showed that after personality manipulation, children with positive personality descriptions were rated as cuter, *SE* = .17, *p* < .001, CI [−1.47, −0.80]. Participants rated children with negative personality descriptions as less cute, *SE* = .24, *p* < .001, CI [1.72, 2.69]. In the control condition, the post-evaluations did not significantly change from the pre-evaluations, *SE* = .11, *p* = .670, CI [−0.18, 0.28].

*Warmth*. The main effect of time on the participants’ ratings of warmth was marginally significant, *F*(1, 71) = 3.52, *p* = .065, η_p_^2^ = .05. Regarding warmth ratings, we found a significant interaction between time and personality descriptions, *F*(2, 142) = 173.79, *p* < .001, η_p_^2^ = .71. After personality manipulation, the warmth ratings increased for children with positive personality descriptions, *SE* = .17, *p* < .001, CI [−2.36, −1.69]. In contrast, children with negative personality descriptions received lower ratings, *SE* = .23, *p* < .001, CI [2.06, 2.97]. In the control condition, the post-evaluation did not change significantly from the pre-evaluations, *SE* = .12, *p* = .369, CI [−0.13, 0.34].

*Competence*. There was a significant main effect of time on participants’ competence ratings, *F*(1, 71) = 13.61, *p* < .001, η_p_^2^ = .16. We found a significant interaction effect between time and personality description, *F*(2, 142) = 81.83, *p* < .001, η_p_^2^ = 0.54. Competence ratings increased after personality manipulation in children with positive personality descriptions, *SE* = .17, *p* < .001, CI [−1.60, −0.94]. In contrast, negative personality descriptions decreased ratings, *SE* = .25, *p* < .001, CI [1.56, 2.55]. Unlike the cuteness and warmth ratings, competence ratings decreased from pre-to post-evaluation in the control condition, *SE* = .12, *p* < .001, CI [0.20, 0.69].

*Infantile characteristics*. Time had a significant main effect on participants’ ratings of infantile characteristics, *F*(1, 71) = 15.09, *p* < .001, η_p_^2^ = .18. As expected, the interaction effect between time and personality description was significant, *F*(2, 142) = 51.04, *p* < .001, η_p_^2^ = .42. In the positive personality condition, participants perceived more infantile characteristics in the child faces after the manipulation, *SE* = .15, *p* = .011, CI [−0.70, −0.10]. In the negative personality condition, participants perceived less infantile characteristics, *SE* = .21, *p* < .001, CI [1.19, 2.02]. In the control condition, the post-evaluation did not change significantly from the pre-evaluations, *SE* = .11, *p* = .763, CI [−0.18, 0.25].

*Mediation predicting nurturing motivation*. Following the steps in establishing mediation [66], we examined correlations among the variables in the mediation model. S1 Table shows the results of the partial correlations with parental status as covariates. All the variables showed significant correlations with each other. Cuteness was highly correlated with perceived infantile characteristics (*r* = .86, *p* < .001).

**Fig 3:**
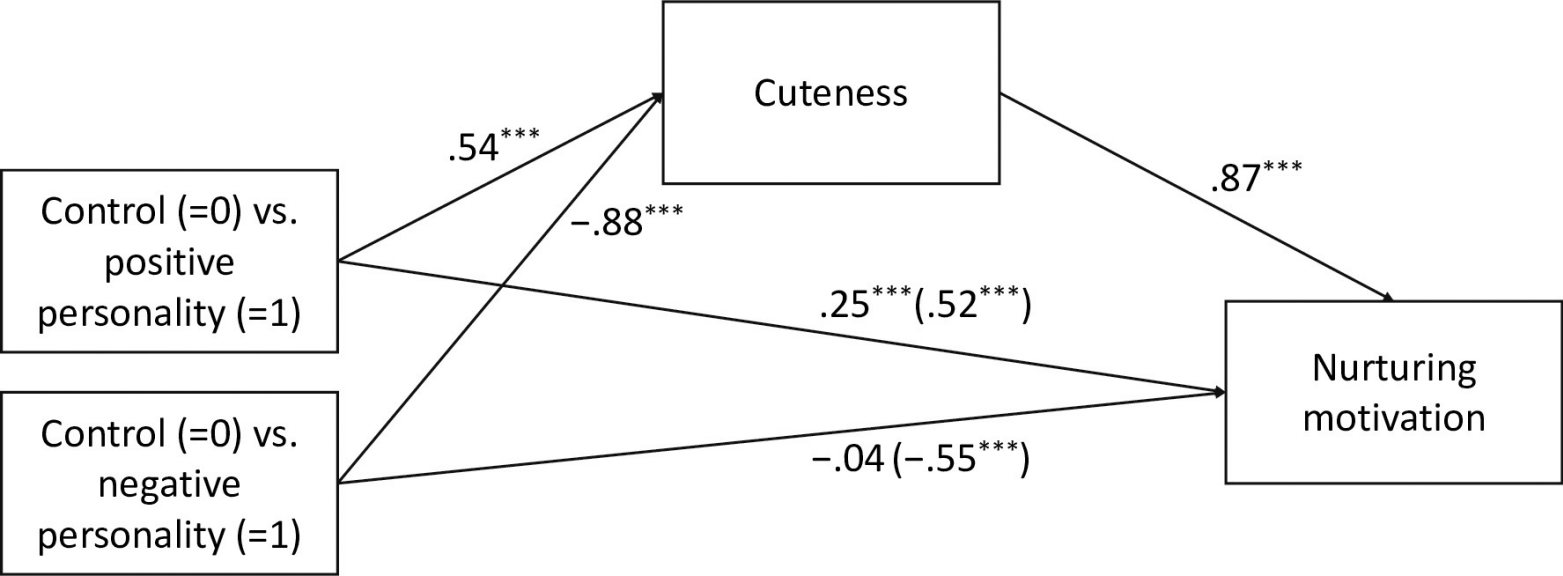
Multi-categorical mediation analysis predicting nurturing motivation (Study 1). *N* = 72. *p < .05, **p < .01, ***p < .001. Regression coefficients were standardized.

Fig 3 presents the results of mediation analysis. In the positive personality condition, the indirect effect of perceived cuteness on nurturing motivation was significant (IE = 0.47, CI [0.24, 0.71]). The direct effect of positive personality descriptions was reduced but remained significant (β = .25, *p* < .001). In the negative personality condition, the indirect effect of perceived cuteness was significant in the opposite direction (IE = −0.77, CI [−0.98, −0.56]). The direct effect of negative personality descriptions was no longer statistically significant (β = −.04, *p* = .521). The proportions of mediated effect (indirect/total effect) were .65 (positive personality descriptions) and .95 (negative personality descriptions), indicating that the indirect pathways account for a high proportion of the effect of personality descriptions on nurturing motivation. The results supported H2, which is that perceived cuteness mediates the link between type of personality description and nurturing motivation toward young children.

For an exploratory analysis (S4 File), we tested the same mediation model with parental status as covariates. Again, the indirect effect of perceived cuteness on nurturing motivation was significant for positive personality descriptions (IE = 0.48, CI [0.23, 0.71]) and negative personality descriptions (IE = −0.78, CI [−0.99, −0.56]). Furthermore, we tested another mediation model with perceived infantile characteristics as mediators. In short, perceived infantile characteristics partially mediated the link between positive and negative personality descriptions and nurturing motivation. The proportions of mediated effect (indirect/total effect) were .43 (positive personality descriptions) and .72 (negative personality descriptions).

### Discussion

Supporting our hypotheses, Study 1 showed that personality descriptions influence young children’s perceived cuteness, warmth, and competence. Perceived cuteness mediates the link between positive and negative personality descriptions and nurturing motivation. The mediation proportion was larger for perceived cuteness than perceived infantile characteristics. This result supports our assumption that nurturing motivation is not only about perceiving infantile characteristics in child faces. Perceived cuteness encompasses the physical and nonphysical traits of children, including their personality.

However, several questions remain, and we will address them in Study 2. First, does the effect of personality descriptions vary according to the child’s initial level of cuteness? In Study 1, we used facial stimuli of children with moderate cuteness based on a pilot study. A study using photographs of adults with varying degrees of physical attractiveness showed that personality descriptions change perceived physical beauty to attractive, unattractive, and neutral targets [11]. Is this true for young children?

Second, does personality descriptions have lasting effects on cuteness and social evaluations (warmth and competence)? Only a few studies in naturalistic settings [17] have examined the long-term impact of personality on perceived attractiveness. Is the effect of experimental manipulation of personality enduring?

Third, does this effect interact with a child’s gender? Although Study 1 did not find any substantial effect of child gender (S2 File), previous research has shown that knowledge of a child’s gender influences perceived physical and nonphysical characteristics, including cuteness [67]. Adults rated labeled boys (children with a label “boy”) as cuter than labeled girls [68]. However, adults usually associate cuteness with female children [69]. Given the complex interplay between gender and cuteness, we examined the effects of personality descriptions on perceived cuteness, warmth, and competence separately in Study 2a (boys) and Study 2b (girls).

## Study 2

### Method

#### Power analysis

Study 1 showed that the effects of personality descriptions on perceived cuteness and other dependent variables under investigation were moderate. G*Power (Version 3.1) [59] indicated that a sample size of 24 would be needed to obtain 80% power to detect a medium effect (*f* = .25) with an alpha of .05 in repeated-measures ANOVA. Considering data loss in the follow-up survey, our goal was to recruit 50 participants for Studies 2a and 2b.

#### Participants

We invited female crowdsourced workers to participate in Studies 2a and 2b. The exclusion criterion for Study 2a and Study 2b was having participated in Study 1.

*Study 2a*. A total of 56 women between 21 and 48 years of age participated in the study (*M*_*age*_ = 32.8, *SD* = 6.6). Of these, 28 participants were parents (50.0%).

*Study 2b*. A total of 52 women aged 20–48 years participated in the study. One participant was excluded because she contacted the investigator in the middle of the survey to avoid following the instructions, leaving 51 participants for data analysis (*M*_*age*_ = 32.2, *SD* = 6.8). Of these, 18 participants were parents (35.5%).

*Follow-up study*. One week later, those who participated in Study 2a or Study 2b were invited to participate in the survey. Among them, 44 participants in Study 2a (78.6%) and 42 participants in Study 2b (82.4%) participated.

#### Materials and procedure

Based on the pilot study described in the section (Materials and methods), we used 18 images of children (nine boys and nine girls) with varying degrees of cuteness (high, moderate, and low) to examine whether physical cuteness moderates the effect of personality descriptions on perceived cuteness, warmth, and competence.

Participants in Study 2a saw nine pictures of boys and participants in Study 2b saw nine images of girls in a counterbalanced order. The procedure for manipulating the personality descriptions and pre-and post-evaluations was the same as in Study 1 except for the numbers of trials and question items. The number of trials in each condition was increased from two to three. Moreover, the two items for measuring nurturing motivation and the other three items (vulnerable, naïve, and caring) for infantile characteristics were not included. This was because the perceived cuteness and infantile characteristics were highly correlated and perceived infantile characteristics explained a smaller proportion of the link between personality descriptions and nurturing motivation as a mediator.

A week later, those who agreed to participate in the follow-up study viewed the same set of nine facial images of boys (Study 2a) or girls (Study 2b) in a counterbalanced order and indicated whether they had seen the face a week before. We included the recognition item to make sure that participants remember the child faces a week later. The recognition rate was high (86.4–100.0%). After the recognition task, they rated the perceived cuteness, warmth, and competence of their children.

### Statistical analysis

To test H1a, H1b, and H3, a series of 2 (time: pre-evaluation, post-evaluation) × 3 (personality descriptions: positive, negative, control) × 3 (physical cuteness: high, medium, low) repeated-measures ANOVA were performed. The dependent variables were cuteness, warmth, and competence. The significance level was set at .05. As in Study 1, we also test the hypotheses, using GLMM (S5 File).

### Results

#### Preliminary analyses

Manipulation of personality descriptions was successful. In line with the results of the pilot test, there was a main effect of personality description (*F*(2, 212) = 779.97, *p* < .001, η_p_^2^ = .88). Participants rated positive personality descriptions (*M* = 7.90) as more positive than negative personality (*M* = 3.02), *t*(106) = 36.25, *SE* = .14, *p* < .001, *d* = 1.39; and neutral descriptions (*M* = 4.88), *t*(106) = 23.78, *SE* = .13, *p* < .001, *d* = 1.31. The Cronbach’s alphas were .92−.95 for cuteness, .86−.91 for warmth, and .85−.90 for competence.

#### Effects of personality descriptions before and after manipulation and after one week

**Fig 4:**
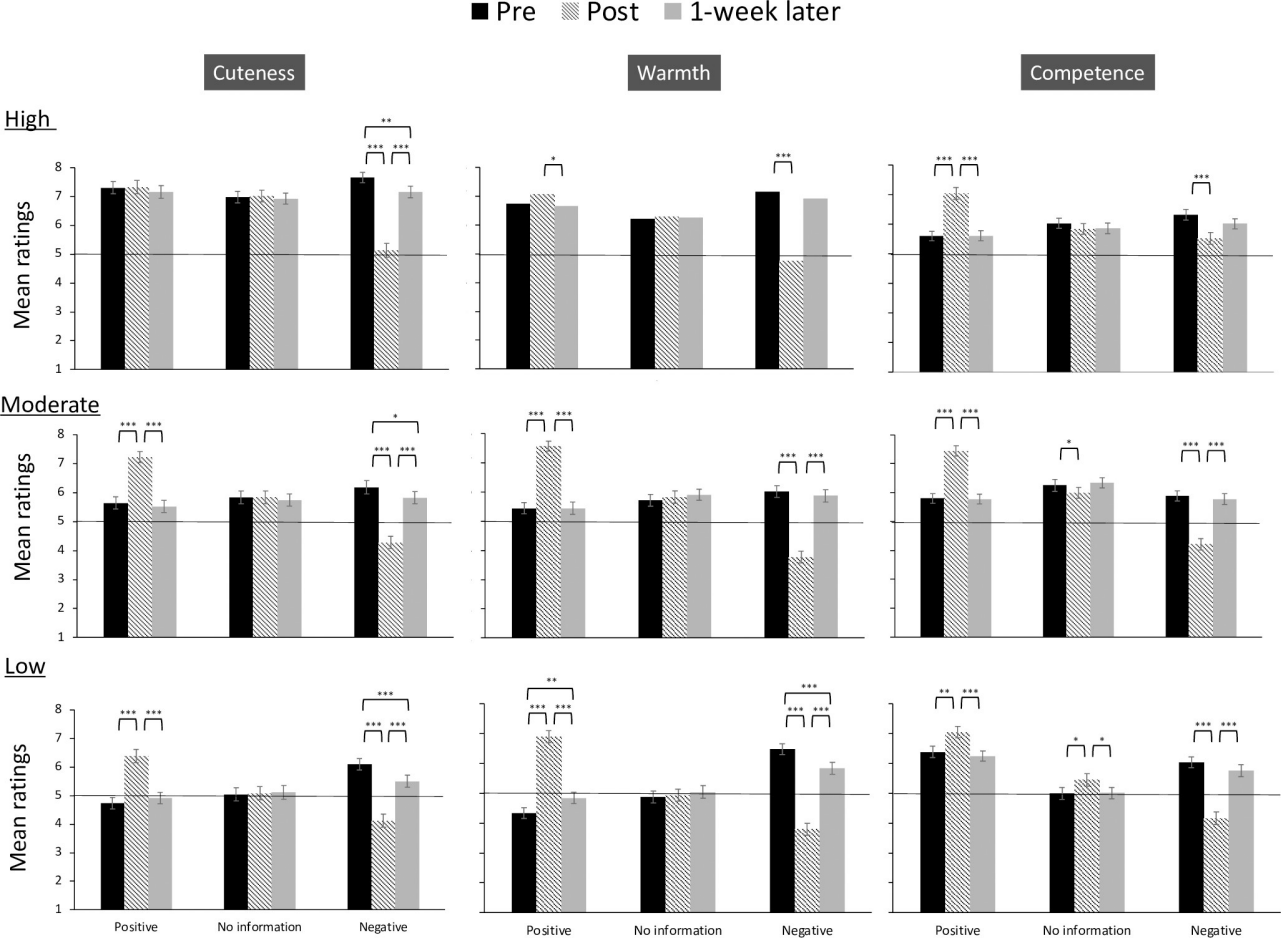
Cuteness, warmth, and competence ratings before/after the personality manipulation and 1 week later (Study 2). Error bars indicate standard error. The dotted lines indicate the midpoints. *p < .05, **p < .01, ***p < .001. High, moderate, and low indicate the levels of physical cuteness.

Fig 4 presents the changes from the pre-evaluation to the after one week evaluation. For children in the negative personality condition, the results partially supported H3, which is that the effect of personality information on perceived cuteness, warmth, and competence will have long-term effects.

*Cuteness*. We did not find a significant interaction effect of time × manipulation × physical cuteness level × child gender, *F*(8, 672) = 1.04, *p* = .407, η_p_^2^ = .01. Similarly, the effect of personality manipulation on the cuteness ratings did not differ significantly by child gender, *F*(4, 336) = 1.13, *p* = .686, η_p_^2^ = .01. For these reasons, we did not consider the interaction effect of child gender thereafter.

There was a significant interaction effect of time × personality descriptions× physical cuteness level, *F*(8, 672) = 6.41, *p* < .001, η_p_^2^ = .07. Positive personality descriptions increased the cuteness ratings (post-evaluation vs. pre-evaluation) for children with moderate and low cuteness. However, the cuteness ratings did not change significantly for high-cute children in the positive personality condition. In the negative personality descriptions, the cuteness ratings decreased significantly for all children.

After a week, the cuteness ratings did not differ significantly from the pre-evaluation for all children in the positive personality condition. However, in the negative personality condition, the cuteness ratings for all children were significantly lower than the pre-evaluation.

*Warmth*. As for cuteness ratings, there was no significant interaction effect of time × manipulation × physical cuteness level × child gender, *F*(8, 672) = 1.23, *p* = .280, η_p_^2^ = .01. The effect of personality manipulation did not differ by child gender, *F*(4, 336) = 1.36, *p* = .248, η_p_^2^ = .02.

There was a significant interaction effect of time × personality descriptions× physical cuteness level, *F*(8, 672) = 14.08, *p* < .001, η_p_^2^ = .14. Positive personality descriptions increased the warmth ratings (post-evaluation vs. pre-evaluation) for children with moderate and low cuteness, but not for high-cute children. In the negative personality descriptions, the warmth ratings decreased significantly for all children.

After a week, the warmth ratings did not differ significantly from the pre-evaluation for high-cute and moderate-cute children in the positive personality condition. The warmth ratings for low-cute children were higher than the pre-evaluation. In the negative personality condition, the warmth ratings for low-cute children were significantly lower than the pre-evaluation.

*Competence*. As for cuteness and warmth ratings, there was no significant interaction effect of time × manipulation × physical cuteness level × child gender, *F*(8, 672) = 1.35, *p* = .216, η_p_^2^ = .02. The effect of personality manipulation did not differ by child gender, *F*(4, 336) = 0.86, *p* = .486, η_p_^2^ = .01.

There was a significant interaction effect of time × personality descriptions× physical cuteness level, *F*(8, 672) = 7.99, *p* < .001, η_p_^2^ = .09. Positive personality descriptions increased the competence ratings (post-evaluation vs. pre-evaluation) for all children, while negative personality descriptions reduced the ratings for all children.

After a week, the competence ratings did not differ significantly from the pre-evaluation for all children in the positive and negative personality conditions.

### Discussion

Study 2 examined whether personality descriptions affect perceived cuteness, warmth, and competence for children independently rated as very cute, moderately cute, and not very cute. The results showed that positive and negative personality descriptions differentially influenced the ratings for high-, moderate-, and low-cute children. Positive personality descriptions had small or no effects on cuteness and warmth ratings for high-cute children. Conversely, negative descriptions consistently reduced the ratings of all children with varying degrees of cuteness. An interesting finding was that the effect of personality descriptions waned after one week. However, there were some exceptions. The effect of negative personality descriptions on cuteness ratings lasted for a week for all children. A similar prolonged effect of negative personality descriptions was found for warmth ratings, but only for low-cute children.

## General discussion

In two pre-registered studies, we demonstrated the effect of personality descriptions on the perceived cuteness, warmth, and competence of young children. Our results showed that, as for adults and infants [11,49], personality is important for evaluating children’s cuteness and social and intellectual competencies. Furthermore, Study 1 showed that personality descriptions influenced female adults’ motivation to care for their children. These findings represent the first known evidence that personality descriptions of young children affect their cuteness, social evaluation, and nurturing motivation toward them.

Our results support the notion that the baby schema effect may not only be about perceiving the infantile characteristics of children [50]. Child characteristics, other than infantile appearance, such as personality traits, influenced parental nurturing motivation. Moreover, these results support prior findings that the perception of cuteness may be shaped by experience, and the target may not have an infantile appearance [70]. Although the baby schema effect underscores the set of infantile appearances of children to activate nurturing motivation and behavior, how people interpret or process information may influence the outcome in tandem.

Consistent with the negativity bias and previous findings on impression formation [6–8], negative personality descriptions consistently reduced cuteness, warmth, and competence ratings for high-, moderate-, and low-cute children. In comparison, the effects of positive personality descriptions on cuteness and warmth ratings were limited to moderate and low-cute children. Attractive children may reap many benefits [5]; however, negative personality descriptions have unfavorable effects on children regardless of their cuteness.

The effect of personality manipulation on cuteness ratings lasted long for all children in the negative personality condition. Moreover, low-cute children in the negative personality condition also received consistently lower warmth ratings after one week. The effect of personality manipulation on competence was short-lived. Cuteness and warmth have two shared components: social engagement and moral concern for the target. Moreover, both cuteness and warmth are validated cross-culturally and promote active helping and nurturing relationships [36,37,71]. Cuteness and warmth may have overlapping qualities, and negative personality information about young children had longer effects on cuteness and warmth, compared to competence.

Our results suggest that negative personality descriptions reduce the baby schema effect on parental motivation for caregiving. This may partially explain why babies and young children who have the most robust baby schema effect are most vulnerable to violence at home [44]. Perceived cuteness in children may change as parents attribute positive or negative personality traits to children. Parents at a high risk of child abuse tend to interpret a child’s intention negatively in an ambiguous situation and give harsh punishments that may escalate into physical abuse [72]. Our results provide a possible explanation for how and why the innate parenting system might become dysfunctional.

### Future directions

Although this study provides novel findings on the malleability of children’s physical attractiveness, some limitations should be noted with directions for future research.

First, we recruited only female participants aged between 20 and 48 years. Previous studies have obtained inconsistent results on whether age, sex, and hormonal levels moderate cuteness perception. Both adults and adolescents prefer children with high baby schema (younger children) over older children, but adolescents rate likability lower than adults did [60]. The effect of the baby schema may be task dependent. Women are more sensitive to baby cuteness than men when asked to choose a cuter baby from a pair of baby faces [73]. However, cute babies are equally rewarding for men and women, as evidenced by their performance on the wanting task, which requires key-pressing to look longer at cute baby faces [74]. In addition, future studies should consider the hormonal status of female participants.

Second, this study and others [49] used facial stimuli from unfamiliar children. One intriguing question is whether mere exposure to a child’s face increases their perceived cuteness. Do adults evaluate familiar children faces cuter than unfamiliar child faces? Indirect evidence [75] suggests that cuteness ratings may increase as people become more familiar with their children. In addition, the interaction may be rewarding for adults. The perceived cuteness of infants increased after adults had repeated positive interactions with them [49]. Future studies should examine whether repeated exposure to a child’s face increases their cuteness ratings and nurturing motivation.

Third, future research is required to explore whether parents’ personality influences the perceived cuteness of their own child and parental motivation for care. The effect of a child’s personality on perceived cuteness and nurturing motivation may differ among parents who evaluate their own child. When parents assess the cuteness of their child, visual cues other than infantile appearance influence the ratings, such as facial resemblance and perceived similarity in personality to their parents [31]. Neuroimaging evidence has shown that parents exhibit different neural responses to their own and unfamiliar children [76,77]. Moreover, the perceived personality traits of their own child may not be fixed, as parents interact with their child daily. Thus, future research should examine how parents’ perceptions of their children’s personality traits influence perceived cuteness and nurturing motivation to test the robustness of the effect of personality.

Fourth, it remains unclear how the impression about a child’s personality is formed and whether the effect of personality information persists or disappears quickly in a naturalistic setting. In this study and others [49], participants made evaluations after a short phrase of learning about personality. However, the status of social relationships is constantly changing, and in real life, learning about personality gradually takes place. Future research should employ a longitudinal design in a naturalistic setting to investigate the long-term effects of personality traits on children’s perceived cuteness and parents’ nurturing motivation.

Finally, future studies should consider demand characteristics of the experimental design. Because the differences in positive and negative personality descriptions were obvious, the participant may have modified her ratings accordingly. Future research is required to consider this experimental artifact when designing the experiment.

## Conclusions

Using photographs of young children’s faces, we demonstrated the effects of personality descriptions on the perceived cuteness, warmth, and competence of young children and nurturing motivation toward them. Although the effect may be short-lived, the baby schema effect may be moderated by the knowledge of the child’s personality traits, even if they are arbitrarily assigned. Children’s infantile cute appearances are appealing to our eyes, but nurturing motivation toward children may depend on the nonphysical traits of children, such as personality.

## Supporting information

S1 Table Partial correlations among variables in the mediation model. (DOCX)

S1 File Pilot 1 and 2. (DOCX)

S2 File Gender differences (Study1). (DOCX)

S3 File Additional analysis for H1a and H1b (Study 1). (DOCX)

S4 File Additional mediation analysis (Study 1). (DOCX)

S5 File Additional analysis for H1a and H1b (Study 2). (DOCX)
