# Supplementary material for: Personality descriptions influence perceived cuteness of children and nurturing motivation toward them
Source: PLoS One. 2023 Jan 18;18(1):e0279985. doi: 10.1371/journal.pone.0279985 (PMC9847979; doi:10.1371/journal.pone.0279985)
Supplement: S1 Table — (DOCX) [file pone.0279985.s001.docx]

**S1 Table. Partial Correlations Among Variables in the Mediation Model**

|  | 3 | 4 | 5 |
| --- | --- | --- | --- |
| 1. Positive personality information | .46^***^  [.35, .57] | .37^***^  [.25, .46] | .53^***^  [.42, .62] |
| 2. Negative personality information | −.54^***^  [−.63, −.45] | −.45^***^  [−.56, −.32] | −.55^***^  [−.65, −.43] |
| 3. Cuteness |  | .86^***^  [.82, .89] | .94^***^  [.92, .96] |
| 4. Perceived infantile characteristics |  |  | .89^***^  [.85, .91] |
| 5. Nurturing motivation |  |  |  |

^***^*p* < .001. *N=* 72. Parental status was entered as a covariate. Positive personality information: control = 0, positive personality condition = 1; Negative personality information: control = 0, negative personality information =1. The range in the square brackets indicates 95% confidence interval.
