# Supplementary material for: Personality descriptions influence perceived cuteness of children and nurturing motivation toward them
Source: PLoS One. 2023 Jan 18;18(1):e0279985. doi: 10.1371/journal.pone.0279985 (PMC9847979; doi:10.1371/journal.pone.0279985)
Supplement: S1 File — (DOCX) [file pone.0279985.s002.docx]

**S1 File. Pilot 1 and 2**

**Pilot 1: Facial images of children**

We collected royalty-free images of preschool children, posing with neutral expression with head pointing straight at the camera. Neutral facial expressions were preferred because neutral expressions evoke stronger nurturing experiences than happy and sad facial expressions [1]. Forty royalty-free images of girls and boys aged 3–4 years were obtained from the Photo AC website (https://www.photo-ac.com/). The images had a 3-year-old or 4-year-old tag and were uploaded by authorized users. Eighteen images were selected for the final set. The age range was chosen because baby schema effects are present beyond infancy [2] and decrease after four and a half years old [3]. The children were posed with neutral expressions and their heads pointed straight at the camera. Because symmetrical bilateral facial features influence facial attractiveness ratings [4], asymmetries (e.g., eye size and eyebrow shape) were corrected using Photoshop. All the images were cropped in a 10.58 cm × 10.58 cm square (background color = hex #e6e2e1) at a resolution of 72 pixels/inch.

**Method and results**

Fifty-four participants aged 20 or older (40.7% female, *Mage* = 38.2, *SD* = 7.0) assisted in research that evaluated the cuteness of 40 images of female and male children’s faces. Facial images were presented in a counterbalanced order. For each image, participants rated the perceived cuteness (1 = *not at all cute* to 10 = *very cute*) and perceived gender (girls, more likely girls, more likely boys, boys) of the child.

Based on the results, children’s faces were divided into three categories: high, moderate, and low. We also categorized children’s faces as boys and girls or ambiguous. A child’s face was classified as a boy if more than 85% of participants identified the child as a boy.

**Pilot 2: List of Desirable/Undesirable Child Personality Traits**

Twenty personality statements about young children were created by drawing on studies that examined child personality traits [5-6]. As ideal child personality traits differ by culture [7], we also included culture-specific desirable and undesirable child personality traits. For example, we included an item involving *amae* (“Acts like a small child and clings to her mother,” “Sometimes pretends to cry to get his parents’ attention”) because Japanese mothers tend to perceive *amae* (a child seeking parental attention and proximity by acting like a baby) as cute and to signal trust [8].

**Methods and Results**

Seventy participants (61.4% female, *Mage* = 20.4, *SD* = 0.7) rated the extent to which personality statements regarding young children were desirable. The instructions read: “Below is a list of personality statements for young children. For each statement, please rate desirability.” Responses were recorded on a 6-point scale (1 = *very undesirable* to 6 = *very desirable*). Based on these results, the desirable and undesirable personality traits were identified. Desirable personality traits included: “Helps her(his) mother voluntarily,” “Is good at drawing and shows her(his) drawings to others proudly,” and “Helps a friend who needs help.” Undesirable personality traits included: “Yells in a grocery store because her(his) mother says she(he) cannot have the snack she(he) wants,” “Cuts in line because she(he) wants to play in the swings,” and “Often gets in a fight and hits her(his) friend.” The average desirability rating was 3.72 (*SD* = 0.98).

**References**

1. Jia YC, Ding FY, Cheng G, Chen J, Zhang W, Lin N, et al. Adults' responses to infant faces: neutral infant facial expressions elicit the strongest baby schema effect. Q J Exp Psychol. 2021; 74(5): 853–871. [doi.org/10.1177/1747021820981862](https://doi.org/10.1177/1747021820981862)
2. Volk AA, Lukjanczuk J, Quinsey, V. L. Perceptions of child facial cues as a function of child age. Evol Psychol. 2007; 5(4): 801–814. doi.org/10.1177/147470490700500409
3. Luo LZ, Lee K, Li H. (2011). Are children’s faces really more appealing that those of adults? Testing the baby schema hypothesis beyond infancy. J Exp Child Psychol. 2011; 110(1): 115–124. doi.org/10.1016/j.jecp.2011.04.002
4. Gangestad SW, Thornhill R, Yeo RA. Facial attractiveness, developmental stability, and fluctuating asymmetry. Ethol Sociobiol. 1994; 15: 73–85. doi.org/10.1016/0162-3095(94)90018-3
5. Halverson CF, Havill VL, Deal JE, Baker SR, Victor JB, Pavlopoulos V, Besevegis E, Wen L. Personality structure as derived from parental ratings of free descriptions of children: The Inventory of Child Individual Differences. J Pers. 2003; 71(6): 995–1026. doi.org/10.1111/1467-6494.7106005
6. Mõttus R, Soto CJ, Slobodskaya HR. Are all kids alike? The magnitude of individual differences in personality characteristics tends to increase from early childhood to early adolescence. Eur J Pers. 2017; 31: 313–328. doi.org/10.1002/per.2107
7. Holden GW, Edwards LA. Parental attitudes toward child rearing: Instruments, issues, and implications. Psychol Bull. 1989; 106(1): 29–58. [doi.org/10.1037/0033-2909.106.1.29](https://doi.org/10.1037/0033-2909.106.1.29)
8. Rothbaum F, Kakinuma M, Nagaoka R, Azuma H. Attachment and amae. J Cross Cult Psychol. 2007; 38: 465–486. doi.org/10.1177/0022022107302315
