# Supplementary material for: Personality descriptions influence perceived cuteness of children and nurturing motivation toward them
Source: PLoS One. 2023 Jan 18;18(1):e0279985. doi: 10.1371/journal.pone.0279985 (PMC9847979; doi:10.1371/journal.pone.0279985)
Supplement: S2 File — (DOCX) [file pone.0279985.s003.docx]

**S2 File. Gender Differences in the Effect of Personality Information on Perceived Cuteness, Warmth, and Competence (Study 1)**

A series of A 2 (time: pre-evaluation, post-evaluation) × 3 (personality information: positive, negative, control/no information) × 2 (child gender: boy, girl) repeated-measures ANOVAs were performed on the cuteness, warmth, and competence ratings.

**Cuteness**

A 2 (time: pre-evaluation, post-evaluation) × 3 (personality information: positive, negative, no information) × 2 (child gender: boy, girl) repeated measures ANOVAs was performed on the cuteness ratings. There were significant interactions among time, personality information, and gender (*F*(2, 142) = 9.77, *p* < .001, partial η^2^ = 0.12). The results of post-hoc comparisons showed that before manipulation, the girl in the positive personality information condition was rated as significantly cuter than the boy (*p* =.001, CI [0.26, 0.98]). Similarly, before manipulation, boys with negative personality information were rated as significantly cuter than girls, *p* < .001, CI [−1.46, −0.55]. Cuteness ratings did not differ significantly between boys and girls in the control condition (*p* = .223, CI [− 0.18, 0.83]). After manipulation, there were no significant differences in the cuteness ratings for the boys and girls in all conditions (positive personality: *p* = .977, CI [−0.32, 0.31]; negative personality: *p* = .176, CI [−0.74, 0.14]; no personality information: *p* = .204, CI [−0.18, 0.83]). Fig 1. presents the gender differences in changes in cuteness ratings as a function of personality information type.


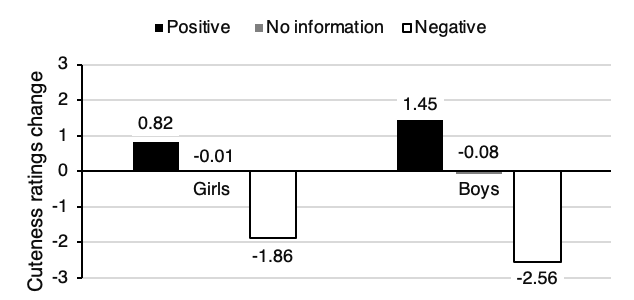


**Fig 1. Gender Differences in Change in Cuteness Ratings.**

**Warmth**

There were significant interactions among time, personality information, and gender (*F*(2, 142) = 12.84, *p* < .001, partial η^2^ = 0.15). Post-hoc comparisons showed that before manipulation, the girl in the positive personality condition was rated as warmer than the boy (*p* =.025, CI [0.05, 0.69]). In the negative personality condition, the boys were rated as warmer than the girls (*p* < .001, CI [−1.77, −.98]). After manipulation, there were no significant differences in the warmth ratings for boys and girls in all conditions (positive personality: *p* = .232, CI [−0.12, 0.47]; negative personality: *p* = .063, CI [−0.55, 0.02]; control: *p* = .055, CI [−0.01, 0.85]). Fig 2. presents the gender differences in changes in warmth ratings as a function of personality information type.


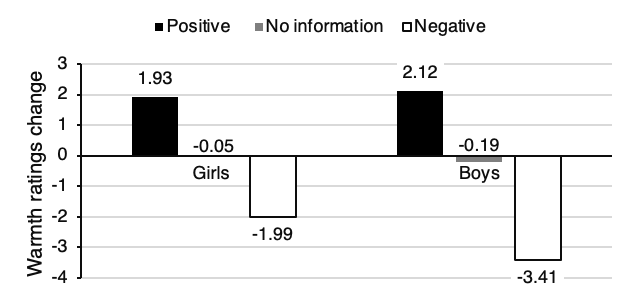


**Fig 2. Gender Differences in Change in Warmth Ratings.**

**Competence**

There were significant interactions among time, personality information, and gender (*F*(2, 142) = 7.03, *p* = .001, partial η^2^ = 0.09). Before manipulation, boys were rated as significantly more or less competent than girls under the following conditions: positive personality, *p* = < .001, CI [1.38, 2.16]; negative personality, *p* < .001, CI [1.24, 2.04]; control, *p* = .036, CI [0.03, 0.81]. After the manipulation, there were still significant differences in competence ratings. Boys in the positive and negative personality conditions were rated as significantly more competent than girls (positive personality: *p* < .001, CI [0.52, 1.12]; negative personality: *p* < .001, CI [0.36, 1.09]). No significant differences in competence ratings were found between boys and girls in the control group, *p* = .113, CI [−0.91, 0.10]. Fig 3 presents the gender differences in changes in competence ratings as a function of personality information type.


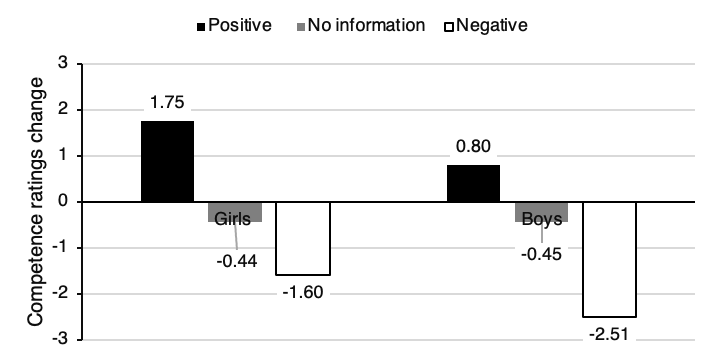


**Fig 3. Gender Differences in Change in Competence Ratings.**

**Perception of Infantile Cuteness**

There were significant interactions among time, personality information, and gender (*F*(2, 142) = 9.82, *p* < .001, partial η^2^ = 0.12). Before manipulation, participants perceived more infantile cuteness in girls than in boys in the positive personality condition (*p* <.001, CI [0.57, 1.21]) and control condition (*p* = .007, CI [0.13, 0.80]). No significant differences in competence ratings were found between boys and girls in the control condition, *p* = .191, CI [−0.57, 0.12]. After manipulation, the gender differences were not significant in the positive, *p* = .687, CI [−0.30, 0.20], and negative personality conditions, *p* = .116, CI [−0.08, 0.67]. The gender difference in the control condition was significant, *p* = .021, CI [0.07, 0.82].

Fig 4 presents gender differences in changes in perceived infantile cuteness in child faces as a function of the personality information type. In the positive personality condition, participants rated the boys as having more infantile characteristics after the manipulation than the pre-manipulation ratings, *p* < .001, CI [0.56, 1.17]. In the negative personality condition, boys and girls received lower ratings than the pre-manipulation ratings at *p* < .001.


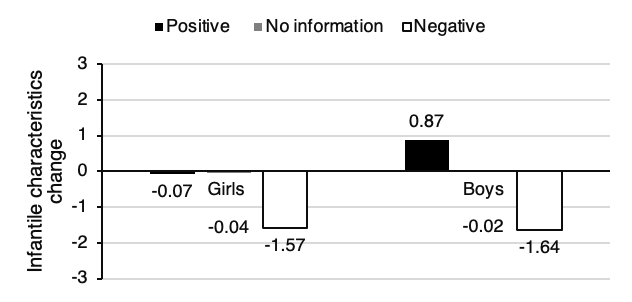


**Fig 4. Gender Differences in Change in Perceived Cuteness
in Child Faces.**

**Gender Differences in the Effects of Personality Information**

For cuteness, there was a significant main effect of personality information for both boys and girls when analyzed separately, *F*(2, 142) = 14.36, *p* < .001, partial *η^2^* = 0.17 for boys, and *F*(2, 142) = 78.45, *p* < .001, partial *η^2^* = 0.525 for girls. For warmth, there was also a significant main effect of personality information for boys, *F*(2, 142) = 32.50, *p* < .001, partial *η^2^* = 0.31, and for girls, *F*(2, 142) = 106.85, *p* < .001, partial *η^2^* = .60. There was also a significant main effect on competence ratings, *F*(2, 142) = 56.05, *p* < .001, *η^2^* = 0.44 for boys and *F*(2, 142) = 85.92, *p* < .001, partial *η^2^* = 0.55 for girls. Finally, for the perception of infantile cuteness, there was a significant main effect of personality information for boys, *F*(2, 142) = 12.81, *p* < .001, partial *η^2^* = 0.15, and *F*(2, 142) = 27.06, *p* < .001, partial *η^2^* = 0.28 for girls. However, the infantile cuteness ratings did not change significantly for girls in the positive personality condition, *p* = .116, CI [−0.31, 0.45].

**Summary**

The overall effects of personality information on perceived cuteness, warmth, and competence were similar among boys and girls, but not perceive infantile cuteness. The effect sizes ranged from 0.17 to 0.44 for boys and from 0.55 to 0.60 for girls. Therefore, the effects of personality information on cuteness, warmth, and competence were large for both sexes. For the perception of infantile cuteness, positive personality information had effects on the boy, but not on the girl.
