# Supplementary material for: Personality descriptions influence perceived cuteness of children and nurturing motivation toward them
Source: PLoS One. 2023 Jan 18;18(1):e0279985. doi: 10.1371/journal.pone.0279985 (PMC9847979; doi:10.1371/journal.pone.0279985)
Supplement: S3 File — (DOCX) [file pone.0279985.s004.docx]

**S3 File. Testing H1a and H1b Using Generalized Linear Mixed-Effect Models (Study 1)**

For additional analyses, we used the lmerTest package for R (3.1-3) [1] to run generalized linear mixed−effect models (GLMM) in R (4.1.2) [2]. We ran GLMM to account for the random effects of participant and facial stimuli. Because we designed this experiment to test the interaction effect of time (pre, post) and personality descriptions (positive, negative, control), we included the random intercepts by participants, participants × time, participants × personality descriptions, and stimuli variability. Fixed effects were time, conditions, and interaction term of time and conditions. The reference levels were the pre- and post-ratings in the control condition. Satterthwaites approximations were used to calculate *p*-values for fixed effects. We coded pre- and post-evaluation as 0 and 1, respectively. As a reference point, we coded the control (no) personality descriptions as 0. For the first dummy variable (D_1_), we coded positive and negative personality descriptions as 1 and 0, respectively. For the second dummy variable (D_2_), we coded positive and negative personality descriptions as 0 and 1, respectively. Below, we report the results of generalized linear mixed models. Tables 1 and 2 show summary tables for fixed effects and random effects respectively. The analytic codes have been shared on https://osf.io/h6rfv/.

**Cuteness**

There was a significant interaction effect between time and positive personality descriptions, b = 3.34, *p* < .001, CI [2.90, 3.78]. The interaction effect of time and negative personality descriptions was also significant, b = 2.16, *p* < .001, CI [1.72, 2.60]. Post-hoc comparisons (Bonferroni) showed that after personality manipulation, children with positive personality descriptions were rated as cuter, *t* = −6.69, *p* < .001, CI [−1.46, −0.80]. Participants rated children with negative personality descriptions as less cute, *t* = 13.06, *p* < .001, CI [1.88, 2.54]. In the control condition, the post-evaluations did not significantly change, *t* = −0.29, *p* = .773, CI [−0.28, 0.38].

**Warmth**

There was a significant interaction effect of time and positive personality descriptions, b = 4.54, *p* < .001, CI [4.15, 4.93]. The interaction effect of time and negative personality descriptions was also significant, b = 2.41, *p* < .001, CI [2.02, 2.80]. After personality manipulation, the warmth ratings increased for children with positive personality descriptions, *t* = −12.94, *p* < .001, CI [−2.33, −1.72]. In contrast, children with negative personality descriptions received lower ratings, *t* = 16.09, *p* < .001, CI [2.21, 2.82]. In the control condition, the post-evaluation did not change significantly, *t* = 0.68, *p* = .498, CI [−0.20, 0.41].

**Competence**

There was a significant interaction effect of time and positive personality descriptions, b = 3.33, *p* < .001, CI [2.90, 3.76]. The interaction effect of time and negative personality descriptions was also significant, b = 1.61, *p* < .001, CI [1.18, 2.04]. Competence ratings increased after personality manipulation in children with positive personality descriptions, *t* = −7.59, *p* < .001, CI [−1.60, −0.94]. In contrast, negative personality descriptions decreased ratings, *t* = 12.25, *p* < .001, CI [1.73, 2.39]. Unlike the cuteness and warmth ratings, competence ratings decreased from pre- to post-evaluation in the control condition, *t* = 2.63, *p* = .001, CI [0.11, 0.77].

**Infantile characteristics**

There was a significant interaction effect of time and positive personality descriptions, b = 2.00, *p* = .018, CI [1.65, 2.35]. The interaction effect of time and negative personality descriptions was also significant, b = 1.57, *p* < .001, CI [1.22, 1.93]. In the positive personality condition, participants perceived more infantile characteristics in the child faces after the manipulation, *t* = −2.66, *p* = .008*,* CI [−0.69, −0.10]. In the negative personality condition, participants perceived less infantile characteristics, *t* = 10.76, *p* < .001, CI [1.31, 1.90]. In the control condition, the post-evaluation did not change significantly, *t* = 0.21, *p* = .828, CI [−0.26, 0.33].

**Table 1. Fixed Effects (Study 1).**

|  | Fixed Effects | | | |
| --- | --- | --- | --- | --- |
|  | Estimate | *SE* | *t* | *p* |
| Cuteness |  |  |  |  |
| Intercept | 6.01 | 0.30 | 19.95 | <.001 |
| Time | −2.21 | 0.17 | −13.06 | <.001 |
| Condition1 (control vs. positive) | −0.13 | 0.35 | −0.37 | .730 |
| Condition2 (control vs. negative) | −0.17 | 0.35 | −0.50 | .645 |
| Time × Condition1 | 3.34 | 0.22 | 14.98 | <.001 |
| Time × Condition2 | 2.16 | 0.22 | 9.68 | <.001 |
|  | Estimate | *SE* | *t* | *p* |
| Warmth |  |  |  |  |
| Intercept | 5.97 | 0.32 | 18.50 | <.001 |
| Time | −2.52 | 0.16 | −16.09 | <.001 |
| Condition1 (control vs. positive) | −0.33 | 0.41 | −0.80 | .470 |
| Condition2 (control vs. negative) | −0.11 | 0.41 | −0.27 | .801 |
| Time × Condition1 | 4.54 | 0.20 | 22.90 | <.001 |
| Time × Condition2 | 2.41 | 0.20 | 12.16 | <.001 |
|  | Estimate | *SE* | *t* | *p* |
| Competence |  |  |  |  |
| Intercept | 5.94 | 0.55 | 10.88 | <.001 |
| Time | −2.06 | 0.17 | −12.25 | <.001 |
| Condition1 (control vs. positive) | 0.17 | 0.74 | 0.23 | .832 |
| Condition2 (control vs. negative) | 0.53 | 0.75 | 0.71 | .529 |
| Time × Condition1 | 3.33 | 0.22 | 15.23 | <.001 |
| Time × Condition2 | 1.61 | 0.22 | 7.38 | <.001 |
|  | Estimate | *SE* | *t* | *p* |
| Infantile Characteristics |  |  |  |  |
| Intercept | 5.74 | 0.25 | 22.74 | <.001 |
| Time | −1.60 | 0.15 | −10.76 | 0.828 |
| Condition1 (control vs. positive) | −0.08 | 0.30 | 0.76 | 0.488 |
| Condition2 (control vs. negative) | −0.31 | 0.30 | −1.03 | 0.358 |
| Time × Condition1 | 2.00 | 0.18 | 11.07 | <.001 |
| Time × Condition2 | 1.57 | 0.18 | 8.70 | <.001 |

Condition1 = control, negative = 0, positive = 1, Condition2 = control, positive = 0, negative = 1.

**Table 2.** **Variance and Standard Deviation for Random Effects (Intercept) Variables.**

|  | Variance | *S.D.* |
| --- | --- | --- |
| Cuteness |  |  |
| Participant | 2.09 | 1.45 |
| Stimulus | 0.09 | 0.30 |
| Participant × Time | 0.13 | 0.37 |
| Participant × Condition1 | 0.02 | 0.14 |
| Participant × Condition2 | 0.22 | 0.47 |
| Residual | 1.79 | 1.34 |
|  | Variance | *S.D.* |
| Warmth |  |  |
| Participant | 1.23 | 1.11 |
| Stimulus | 0.14 | 0.38 |
| Participant × Time | 0.17 | 0.42 |
| Participant × Condition | 0.23 | 0.48 |
| Residual | 1.41 | 1.19 |
|  | Variance | *S.D.* |
| Competence |  |  |
| Participant | 1.29 | 1.14 |
| Stimulus | 0.53 | 0.73 |
| Participant × Time | 0.15 | 0.39 |
| Participant × Condition | 0.15 | 0.39 |
| Residual | 1.72 | 1.31 |
| Infantile Characteristics | Variance | *S.D.* |
| Participant | 1.14 | 1.07 |
| Stimulus | 0.07 | 0.26 |
| Participant × Time | 0.22 | 0.46 |
| Participant × Condition | 0.24 | 0.49 |
| Residual | 1.18 | 1.08 |

Condition1 = control, negative = 0, positive = 1, Condition2 = control, positive = 0, negative = 1.

**References**

1. Kuznetsova A, Brockhoff PB, Christensen RHB. lmerTest Package: Tests in linear mixed effects models. J Stat Softw. 2017; 82(13): 1–26. [doi.org/10.18637/jss.v082.i13](https://doi.org/10.18637/jss.v082.i13)
2. R-project.org. 2022. R: The R Project for Statistical Computing. [online] Available at: https://www.R-project.org/ [Accessed 12 October 2022].
