# Supplementary material for: Personality descriptions influence perceived cuteness of children and nurturing motivation toward them
Source: PLoS One. 2023 Jan 18;18(1):e0279985. doi: 10.1371/journal.pone.0279985 (PMC9847979; doi:10.1371/journal.pone.0279985)
Supplement: S4 File — (DOCX) [file pone.0279985.s005.docx]

**S4 File. Multi-Categorical Mediation Analysis Predicting Nurturing Motivation (Study1).**

Fig 1 presents the results of mediation analysis. In the positive personality condition, the indirect effect of perceived cuteness on nurturing motivation was significant (IE = 0.48, CI [0.23, 0.71]). The direct effect of positive personality descriptions was reduced but remained significant (β = .23, *p* = .001). In the negative personality condition, the indirect effect of perceived cuteness was significant in the opposite direction (IE = −0.78, CI [−0.99, −0.56]). The direct effect of positive personality descriptions was no longer statistically significant (β = −.03, *p* = .656). The proportions of mediated effect (indirect/total effect) were .68 (positive personality descriptions) and .97 (negative personality descriptions), indicating that the indirect pathways account for a high proportion of the effect of personality descriptions on nurturing motivation.

**Fig 1**. Multi-Categorical Mediation Analysis Predicting Nurturing Motivation (Study 1). *N* = 72. ^**^*p* < .01, ^***^*p* < .001. Parental status was used as a covariate. Regression coefficients are standardized. The regression coefficient in brackets indicates a direct effect of positive/negative personality information on nurturing motivation, without the indirect effect of cuteness.

Fig 2 presents the results of multi-categorical mediation. In the positive personality condition, the indirect effect of perceived infantile characteristics on nurturing motivation was significant (IE = 0.30, CI [0.09, –0.50]. The direct effect of positive personality information was reduced, but was still significant after accounting for the indirect effect. In the negative personality condition, the indirect effect of perceived infantile characteristics was significant in the opposite direction (IE = −0.58, CI [−0.80, −0.36]). The direct effect of positive personality information was reduced, but remained significant after accounting for the indirect effect. Therefore, perceived infantile characteristics partially mediate the link between positive/negative personality information and nurturing motivation.

**Fig2. Multi-Categorical Mediation Analysis Predicting Nurturing Motivation (Study 1).**

*N* = 72. ^*^*p* < .05, ^**^*p* < .01, ^***^*p* < .001. Parental status was used as a covariate. Regression coefficients are standardized. The regression coefficient in brackets indicates a direct effect of positive/negative personality information on nurturing motivation, without the indirect effect of perceived infantile cuteness.
