# Supplementary material for: Personality descriptions influence perceived cuteness of children and nurturing motivation toward them
Source: PLoS One. 2023 Jan 18;18(1):e0279985. doi: 10.1371/journal.pone.0279985 (PMC9847979; doi:10.1371/journal.pone.0279985)
Supplement: S5 File — (DOCX) [file pone.0279985.s006.docx]

**S5 File. Testing H1a and H1b Using Linear Mixed Models (Study 2)**

As in Study 1 (S3 File), we used generalized linear mixed−effect models (GLMM) to test whether physical cuteness influenced the effect of personality descriptions on perceived cuteness, warmth, and competence ratings. Participant and facial stimuli were random effects. Time (pre, post, and 1-week later), personality descriptions, and physical cuteness levels were fixed effects.

**Cuteness**

Fig 1 shows fixed effects model for the cuteness ratings. There was a significant interaction effect of time (pre vs. post) and positive personality descriptions, b = 3.61, *p* < .001, CI [3.06, 4.16]. The interaction effect of time and negative personality descriptions was also significant, b = 2.01, *p* < .001, CI [1.46, 2.56]. Post-hoc comparisons (Bonferroni) showed that after personality manipulation, children with positive personality descriptions were rated as cuter, *t* = −9.20, *p* < .001, CI [−1.31, −0.85]. Children with negative personality descriptions were rated as less cute, *t* = 18.12, *p* < .001, CI [1.90, 2.36]. We found no significant change for children in the control condition, *t* = −0.22, *p* = .824, CI [−0.26, 0.20]

**
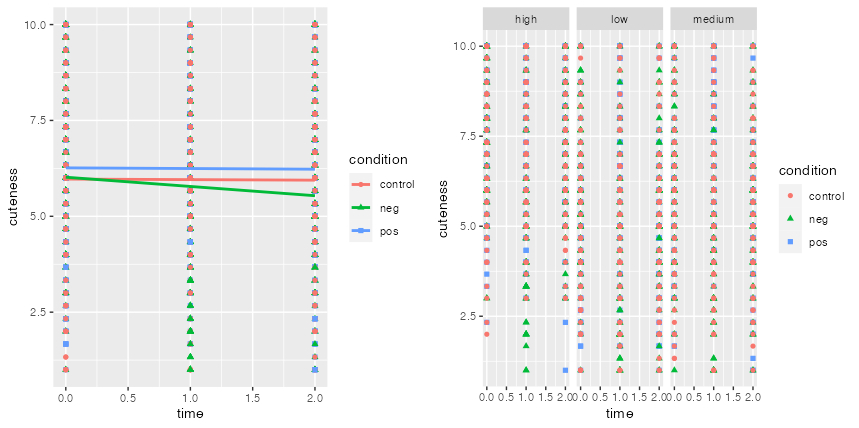
**

**Figure 1. Fixed Effects Model for Cuteness Ratings (Study 2).**

After a week, the cuteness ratings for children with positive personality descriptions did not significantly differ from the pre-evaluation, *t* = 0.30, *p* = .766, CI [−0.20, 0.27]. As expected, the cuteness ratings for children in the control condition did not differ from the pre-evaluation, *t* = 0,24, *p* = .810, CI [−0.20, 0.26]. However, children with negative personality descriptions were rated as less cute than the pre-evaluation, *t* = 4.10, *p* < .001, CI [0.25, 0.71]. Specifically, the cuteness ratings for low-cute children were significantly lower than the pre-evaluation, *t* = 2.94, *p* = .003, CI [0.20, 0.99].

**Warmth**

Fig 2 shows fixed effects model for the warmth ratings. There was a significant interaction effect of time and positive personality descriptions, b = 5.25, *p* < .001, CI [4.71, 5.79]. The interaction effect of time and negative personality descriptions was also significant, b = 2.75, *p* < .001, CI [2.21, 3.28]. Post-hoc comparisons showed that after personality manipulation, children with positive personality descriptions were rated as warmer, *t* = −13.92, *p* < .001, CI [−1.91, −1.44]. Children with negative personality descriptions were rated as less warm, *t* = 20.39, *p* < .001, CI [2.21, 2.68]. The warmth ratings for children in the control condition did not differ from the pre-evaluation, *t* = −0.70, *p* = .484, CI [−0.32, 0.15].

After a week, the warmth ratings did not differ significantly from the pre-evaluation for children in the positive personality condition, *t* = −1.23, *p* = .220, CI [−0.38, 0.27]. In the negative personality condition, the warmth ratings for low-cute children were significantly lower than the pre-evaluation, *t* = 2.84, *p* = .005, CI [0.11, 0.58]. Specifically, the warmth ratings for low−cute children were significantly lower than the pre-evaluation, *t* = 3.17, *p* = .001, CI [0.24, 1.02].


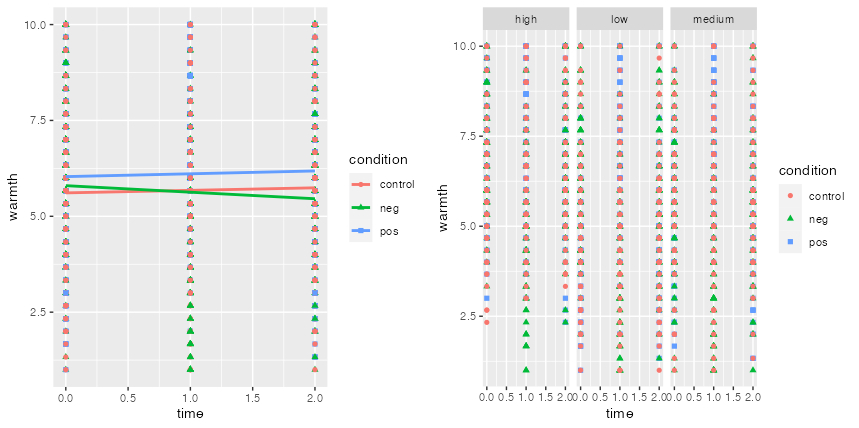


**Fig 2. Fixed Effects Model for Warmth Ratings (Study 2).**

**Competence**

Fig 3 shows fixed effects model for the warmth ratings. The interaction effect of time and positive personality descriptions was significant, b = 2.53, *p* < .001, CI [1.98, 3.08]. The interaction effect of time and negative personality descriptions was also significant, b = 2.33, *p* < .001, CI [1.78, 2.88]. Post-hoc comparisons showed that after personality manipulation, children with positive personality descriptions were rated as more competent, *t* = −9.95, *p* < .001, CI [−1.49, −1.00]. Children with negative personality descriptions were rated as less competent, *t* = 11.60, *p* < .001, CI [1.21, 1.70]. The competence ratings for children in the control condition did not differ from the pre-evaluation, *t* = −0.04, *p* = .484, CI [−0.25, 0.24].

After a week, the competence ratings did not differ significantly from the pre-evaluation for children in the positive personality condition, *t* = 0.39, *p* = .696, CI [−0.20, 0.29], and negative personality condition, *t* =1.84, *p* = .066, CI [−0.02, 0.48].


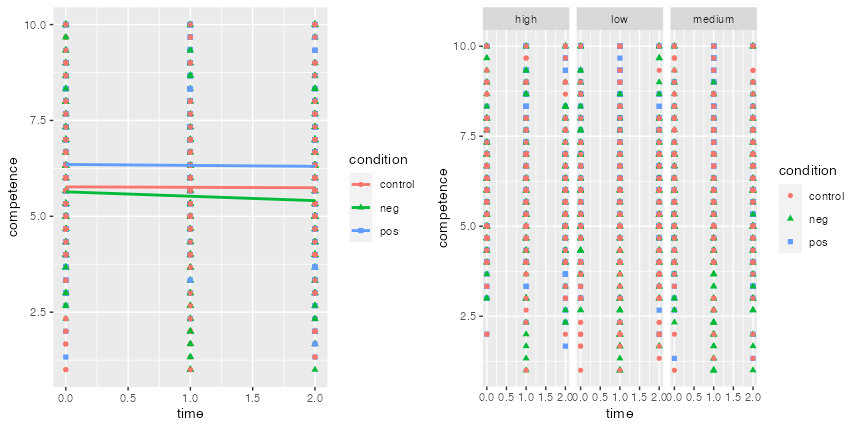


**Fig 3. Fixed Effects Model for Competence Ratings (Study 2).**
